# A mechanistic study on the cellular uptake, intracellular trafficking, and antisense gene regulation of bottlebrush polymer-conjugated oligonucleotides†

**DOI:** 10.1039/d2cb00149g

**Published:** 2022-11-08

**Authors:** Lei Zhang, Yuyan Wang, Peiru Chen, Dali Wang, Tingyu Sun, Zheyu Zhang, Ruimeng Wang, Xi Kang, Yang Fang, Hao Lu, Jiansong Cai, Mengqi Ren, Sijia S. Dong, Ke Zhang

**Affiliations:** a Chemicobiology and Functional Materials Institute, School of Chemistry and Chemical Engineering, Nanjing University of Science and Technology Nanjing 210094 P. R. China; b Department of Chemistry and Chemical Biology, Northeastern University Boston Massachusetts 02115 USA s.dong@northeastern.edu k.zhang@northeastern.edu; c Departments of Chemical Engineering and Bioengineering, Northeastern University Boston Massachusetts 02115 USA

## Abstract

We have developed a non-cationic transfection vector in the form of bottlebrush polymer-antisense oligonucleotide (ASO) conjugates. Termed pacDNA (polymer-assisted compaction of DNA), these agents show improved biopharmaceutical characteristics and antisense potency *in vivo* while suppressing non-antisense side effects. Nonetheless, there still is a lack of the mechanistic understanding of the cellular uptake, subcellular trafficking, and gene knockdown with pacDNA. Here, we show that the pacDNA enters human non-small cell lung cancer cells (NCI-H358) predominantly by scavenger receptor-mediated endocytosis and macropinocytosis and trafficks *via* the endolysosomal pathway within the cell. The pacDNA significantly reduces a target gene expression (KRAS) in the protein level but not in the mRNA level, despite that the transfection of certain free ASOs causes ribonuclease H1 (RNase H)-dependent degradation of KRAS mRNA. In addition, the antisense activity of pacDNA is independent of ASO chemical modification, suggesting that the pacDNA always functions as a steric blocker.

## Abstract 2

We show that a bottlebrush polymer-antisense oligonucleotide conjugate (termed pacDNA) enters cancer cells by scavenger receptor-mediated endocytosis and micropinocytosis, trafficks *via* the endolysosomal pathway, and functions as a steric blocker.

## Graphical abstract

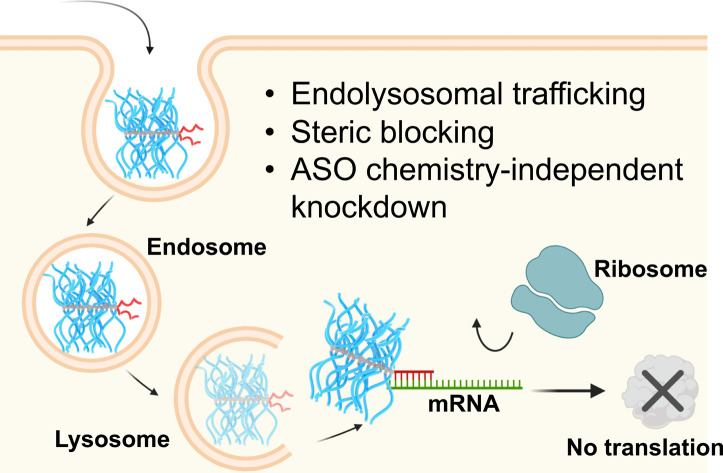

Antisense oligonucleotides (ASOs) are widely used in RNA-directed therapies. To date, several ASOs have been approved by the US Food and Drug Administration (FDA) to treat genetic disorders, metabolic diseases, or viral infection, including fomivirsen, mipomersen, nusinersen, eteplirsen, inotersen, golodirsen, and casimersen,^1–3^ with many more in clinical trials. ASOs can modify the expression of the target gene by altering pre-mRNA splicing, degrading mRNA *via* RNase H, inhibiting translation by steric blocking, and by affecting non-coding RNAs involved in transcriptional and epigenetic regulation.^4–7^

To guarantee that sufficient ASO molecules reach their cytoplasmic or nuclear targets before being degraded, multiple types of chemical modifications including those of the nucleobase, internucleotide linkage, and the pentose, as well as complete backbone replacements have been developed.^8–10^ Still, naked ASOs can be cleared rapidly *in vivo*, leading to poor cellular utilization, increased dosage requirement/cost, and a narrower therapeutic window.^11^ Intracellular delivery systems, which are typically cationic materials (*e.g.*, polymers, peptides, nanoparticles, lipids, ligands, *etc.*),^12–15^ have been developed to promote cellular uptake and endosomal escape. However, delivery vectors often face a difficult dilemma: features that make cellular transfection more efficient, such as the presence of multiple cationic and hydrophobic groups, often lead to increased toxicity and/or poorer pharmacological properties.^16,17^ Thus, current efforts in carrier design have often focused on optimizing transfection efficiency within an acceptable toxicity range.

Our group has focused on a different approach to the *in vivo* efficacy problem by prioritizing the pharmacological properties of the carrier.^18^ The rationale is that, if one can substantially reduce renal clearance, improve plasma pharmacokinetics, and prolong tissue retention, the overall antisense activity *in vivo* can still be greatly enhanced even though the transfection efficiency on the cellular level is moderate. One implication of this philosophy in vector design is that one may do away with polycationic species that drives toxicity, and instead adopt more biologically benign materials that promote blood retention, such as poly(ethylene glycol) (PEG) and zwitterionic polymers.^19,20^

Recently, we have developed a novel form of the ASO vector, termed pacDNA (polymer-assisted compaction of DNA), which consists of a bottlebrush polymer with a multitude of PEG side chains and covalently conjugated ASO strands on the polymer backbone (Scheme 1).^21,22^ The dense arrangement of the PEG side chains endows the ASO with steric selectivity: ASO interactions with proteins are greatly reduced, while hybridization is unaffected in both the kinetic and the thermodynamic sense.^23,24^ Such selectivity is absent with linear PEG-ASO conjugates and drastically reduces enzymatic degradation and most side effects stemming from specific or non-specific DNA-protein interactions, such as coagulopathy and unwanted innate immune system activation.^25^ Importantly, the pacDNA is significantly more persistent in blood, with a 20–50× increase in the elimination half-life and 1–2 orders of magnitude increase in the area-under-the-curve (AUC_∞_) compared with free DNA. The pharmacological improvements result in substantially enhanced ASO activity *in vivo* in several tumor xenograft models.^26^ In one example where we compared pacDNA with AZD4785, a clinical ASO targeting wild-type KRAS mRNA, pacDNA achieved more pronounced tumor suppression levels than AZD4785 at a fraction (2.5%) of the dosage and with greatly reduced dosing frequency in non-small cell lung carcinoma (NSCLC) mouse models.^27^ In addition, the treatment was free of common deleterious effects such as acute toxicity, inflammation, coagulopathy, and anti-PEG immunity.^27^

**Scheme 1:**
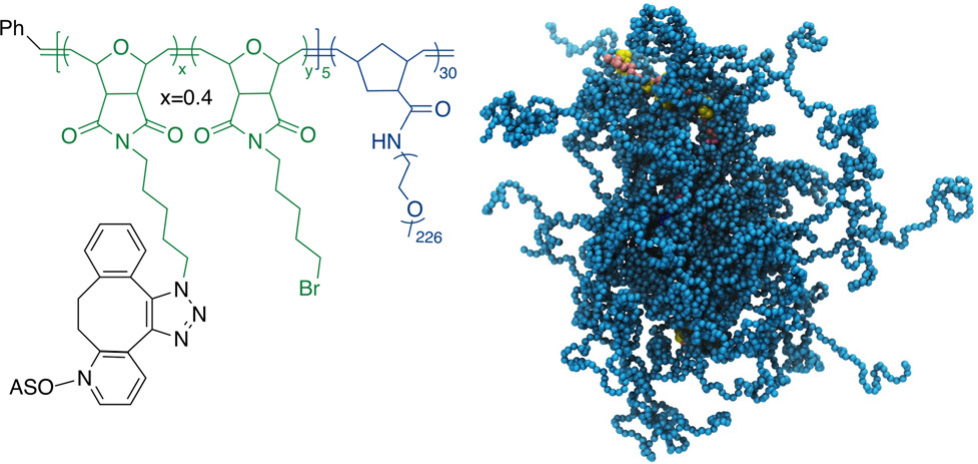
Chemical structure (left) and a coarse-grained molecular dynamics simulation (right) of the pacDNA. In the simulation image, pink/yellow: DNA, cyan: PEG.

When tested *in vitro*, the pacDNA exhibits moderate cellular uptake and can regulate mRNA expression with reasonable efficiency if equipped with an appropriate ASO or siRNA. However, to date, the mechanism for the cellular uptake of the pacDNA and how it regulates protein expression at the molecular level is not well understood. In this study, we explore these aspects using NCI-H358 cells, a human NSCLC cell line harboring the *KRAS*^G12C^ mutation (Fig. 1A). We anticipate that a mechanistic understanding of the uptake, intracellular trafficking, and gene regulation at the cellular level can not only inform future optimizations of the pacDNA platform but also enable completely novel vector designs based on the *in vivo*-first approach.

**Fig. 1:**
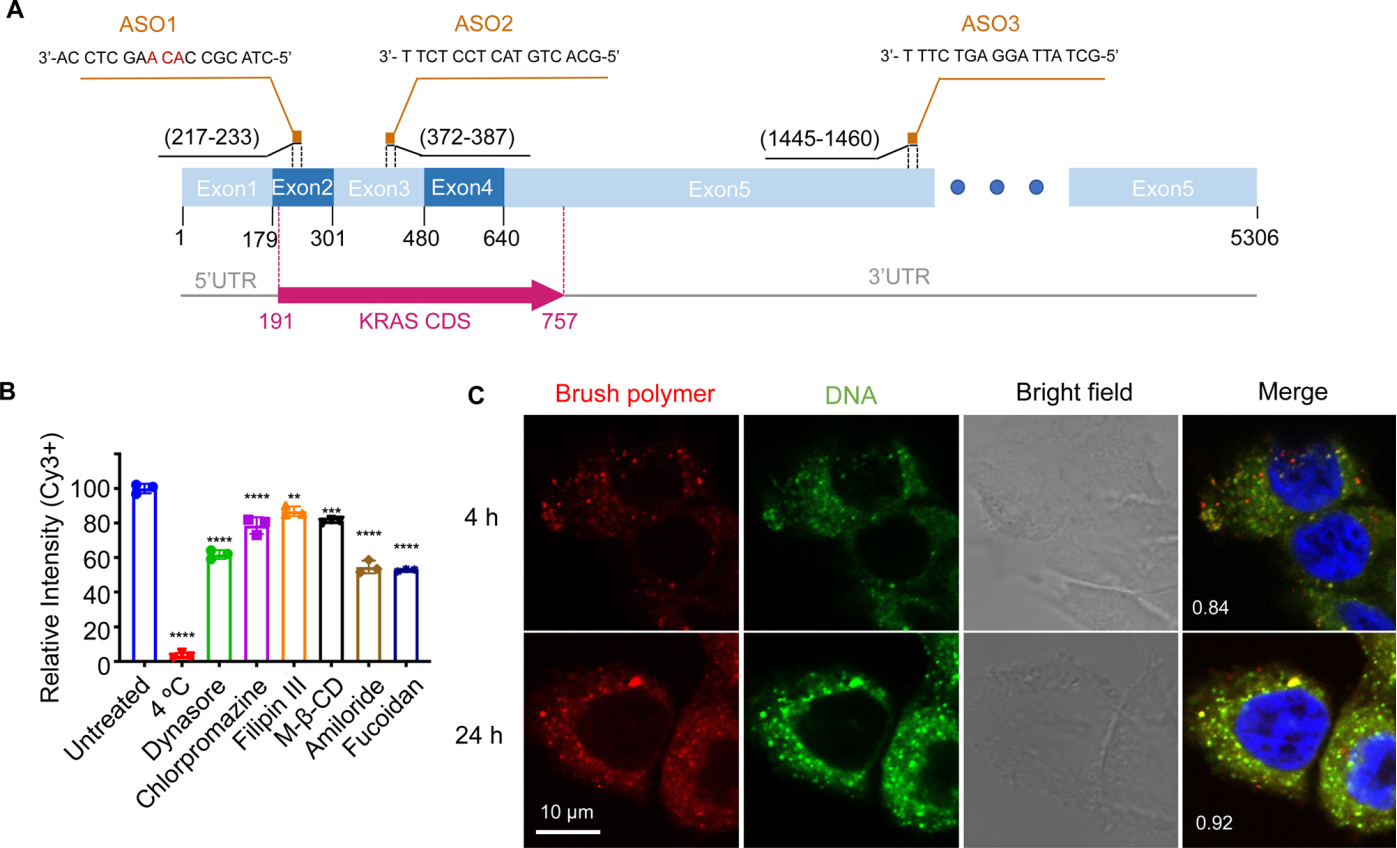
(A) Sequences and binding regions of ASO1, ASO2, and ASO3 to the KRAS transcript isoform b (NM_004985.5). CDS: coding sequence. (B) Uptake of pacDNA by NCI-H358 cells in the presence of various pharmacological blockers. (C) Confocal microscopy of NCI-H358 cells after incubation with dual-labeled pacDNA (red: Cy5-labeled brush polymer; green: Cy3-labeled DNA) for 4 and 24 h. The Manders’ colocalization coefficient is shown in the merged images. The cell nuclei are counter-stained with DAPI and shown in blue.

The pacDNA used in this study consists on average of ∼30 PEG side chains (10 kDa each) and two ASO strands per molecule, and is synthesized according to prior literature (Scheme S1, ESI†).^28^ Table S1 (ESI†) provides a summary of the sample nomenclature, ASO sequence, chemical modifications (if any), and assays performed. To study the mechanism of the cellular uptake of pacDNA in NCI-H358 cells, we pre-treated the cells with different endocytosis inhibitors (Table S2, ESI†) in serum-deprived RPMI-1640 medium for 1 h to block the key pathways of cellular uptake, including lipid raft/caveolae-mediated endocytosis, clathrin-mediated endocytosis, dynamin-mediated endocytosis, and pinocytosis. Next, the pre-treated cells were incubated with Cy3-labeled pacDNA containing a phosphodiester (PO) ASO3 (PO pacDNA-3-Cy3) in serum-free medium for 4 h. Flow cytometry analysis revealed depressed fluorescence intensity for cells treated with low temperature (4 °C), dynasore (inhibitor of dynamin^29^), amiloride (inhibitor of epithelial sodium channel, ENaC^30^), and fucoidan (a competitive ligand for scavenger receptor Class A, SR-A^31^), with reductions of ∼97%, ∼38%, ∼45%, and ∼47%, respectively (Fig. 1B, Fig. S1A and S1B, ESI†). The significant reduction in the cellular uptake at 4 °C indicates that the uptake is predominantly an energy-dependent process as opposed to passive transmembrane diffusion. Sensitivity to amiloride suggests that macropinocytosis plays an important role in the uptake of pacDNA in NCI-H358 cells. Indeed, previous studies report that mutations in Ras proteins can stimulate macropinocytosis in order for cells to use proteins as an amino acid supply.^32^ Cellular uptake is also dependent on dynamin to some extent, which is involved in caveolae- and clathrin-dependent endocytosis. To differentiate the two pathways, cells were treated with filipin III and methyl-β-cyclodextrin (m-β-CD). Both molecules can disrupt the structure of the lipid raft by interfering with cellular cholesterol,^33,34^ which is required for the formation of caveolae.^35^ The treatment resulted in ∼13% and ∼19% reduction in the cell uptake, respectively. On the other hand, treatment with chlorpromazine, which inhibits clathrin-mediated endocytosis by disrupting the assembly and disassembly of clathrin lattices,^36^ reduced the cellular uptake by 21%. SR-A is likely a main receptor for the endocytosis, as treatment with fucoidan blocks roughly half of the uptake, which is coincidentally comparable to the combined contribution of clathrin- and caveolae-dependent endocytosis. SR-A is known to bind to the negatively charged oligonucleotides with its positively charged groove in the collagenous domain.^37^ However, because free DNA is not taken up in high quantities by NCI-H358 cells, it is conceivable that pacDNA improves the adsorption of the DNA onto the plasma membrane, possibly mediated by PEG-cation-membrane coulombic interactions and van der Waals interactions between the hydrophobic polymer backbone and the membrane,^38,39^ leading to more facile SR-A binding to the DNA. Indeed, an anionic form of pacDNA with a negatively charged, hydrophilic backbone undergoes very limited cellular uptake, similar to that of free DNA.^40^ Together, these data identify SR-A-mediated endocytosis (both clathrin- and caveolae-dependent pathways) and macropinocytosis as the main mechanisms for the uptake of pacDNA by NCI-H358 cells. We have previously found that pacDNA with the full phosphothioate modifications (PS pacDNA) enters NCI-H358 cells *via* similar mechanisms, although PO and PS ASOs enter cells very differently without polymer content,^27^ suggesting that the polymer is dominating the uptake mechanism. Because the pacDNA has two covalently linked components, *i.e.* the bottlebrush polymer and the oligonucleotide, we first studied whether the two components remain colocalized. To do so, we labeled the bottlebrush polymer with Cy5 and the oligonucleotide with Cy3 (PO Cy5-pacDNA-3-Cy3) (Scheme S2, ESI†). The dual-labeled pacDNA was incubated with cells in serum-containing medium, and the co-localizations of the two signals were studied using confocal microscopy. The fluorescence images show punctate patterns in the fluorescence signals, suggesting that the majority of pacDNA remains in endosomal structures. The Cy3 (green) and Cy5 (red) signals are highly colocalized 4 h and 24 h post incubation, with the Manders’ colocalization coefficient of 0.84 and 0.92, respectively (Fig. 1C). There are a small number of endosomal patterns where signal colocalization is very poor (red dots), which is likely caused by the movement of those endosomes out of the focal plane between the capture of the Cy3- and Cy5-channel signals. These results suggest that the pacDNA remains largely intact inside the cells for at least 24 h.^21,28^

The structural stability of the pacDNA in cells indicates that signals from the Cy3 component (DNA) is representative of the intracellular localization of both pacDNA components. To study intracellular trafficking following endocytosis, we incubated NCI-H358 cells with Cy3-labeled pacDNA (PO pacDNA-3-Cy3) for different durations of time, followed by immunofluorescence staining to determine their intracellular locations in relation to various protein markers (Fig. 2), including early endosome antigen 1 (EEA1, early endosome),^41^ Ras-related protein 9 (Rab9, late endosome),^42^ and lysosomal-associated membrane protein 1 (LAMP1, lysosome).^43^ After 1 h of incubation, pacDNA colocalizes predominantly with early endosomes (Manders’ colocalization coefficient of 0.57) and to a lesser extent, with late endosomes or lysosomes (Manders’ colocalization coefficient of 0.46 and 0.16, respectively). Colocalization with late endosome increased slightly to 0.64 (4 h) and stayed relatively unchanged over the course of 24 h, while lysosomal colocalization increased more pronouncedly (Manders’ colocalization coefficient of 0.75 at 24 h). We performed the same set of experiments under serum-deprived conditions, and pacDNA exhibited a similar trafficking pathway (Fig. S2A and S2B, ESI†). Collectively, pacDNA primarily adopts the conventional endolysosomal route of trafficking after entering NCI-H358 cells, irrespective of the presence of serum proteins in the culture media.

**Fig. 2:**
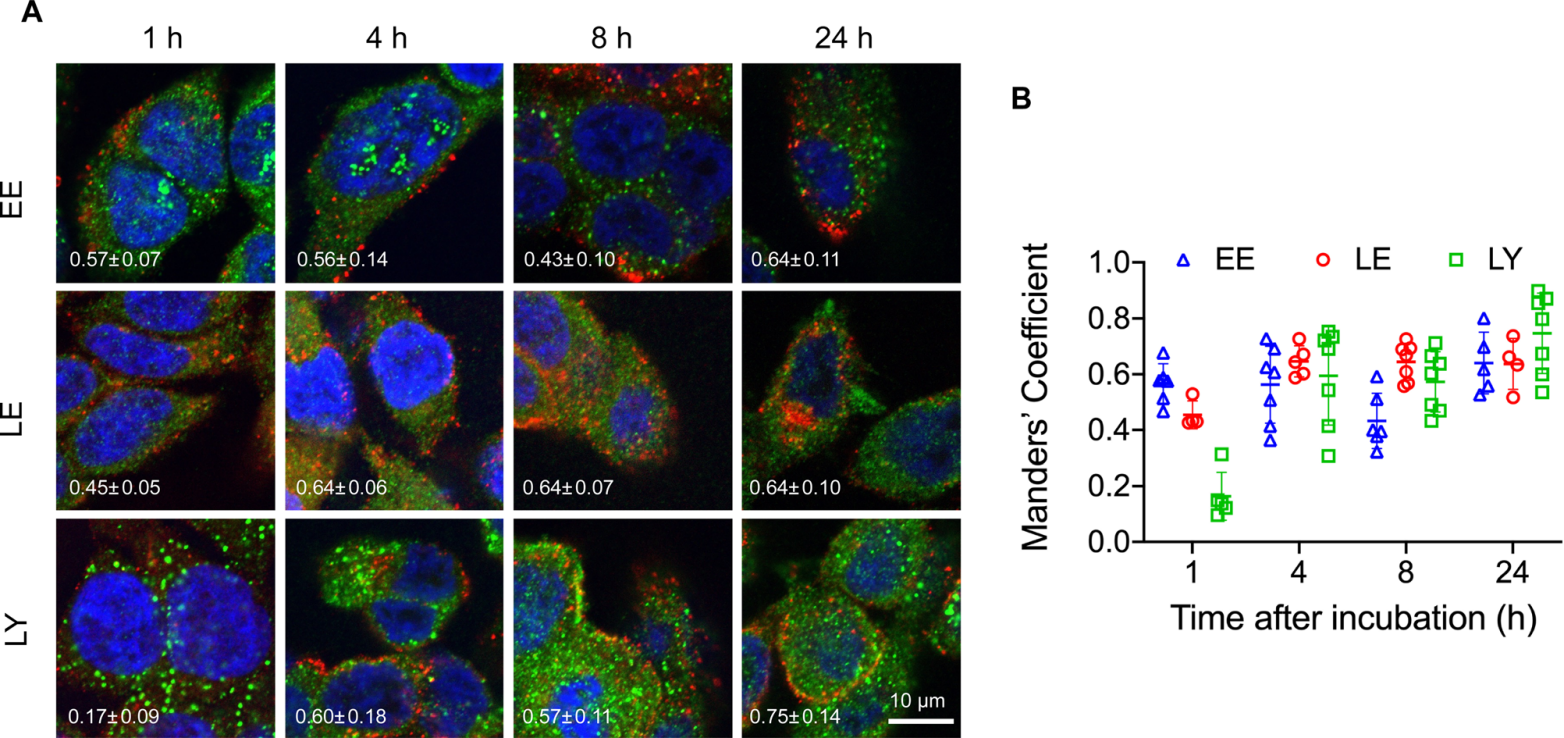
(A) Intracellular trafficking of pacDNA in NCI-H358 cells following different durations of incubation. pacDNA: red; immunofluorescence staining of organelle markers: green. The markers include EEA1 (early endosome, EE), Rab9 (late endosome, LE), and LAMP1 (lysosome, LY). Average Manders’ colocalization coefficient with standard deviation (S.D.), shown in the bottom left side of each image, of 0.5 or above indicates substantial colocalization. (B) Summary of the Manders’ colocalization coefficient calculated from confocal images. In each treatment, over 20 cells in at least five images were analyzed, where each data point represents one image collected.

To probe how the pacDNA inhibits the expression of target protein, we selected three antisense ASOs that have been shown effective in lowering KRAS mRNA. These ASOs target the G12C mutation site in exon 2 (ASO1),^44^ a bulge in exon 3 (ASO2),^45^ and a segment in the 3′ UTR of KRAS mRNA (ASO3, Fig. 1A and Table S1, ESI†).^46^ We first confirmed the antisense activity of the free ASOs with full PS modifications. Lipofectamine2k (Lipo), a cationic liposomal transfection agent, was used to form lipoplexes with the ASOs, which were incubated with NCI-H358 cells for 24 h before mRNA levels were determined by quantitative real-time polymerase chain reaction (qRT-PCR) (Fig. 3A). All three ASOs showed apparent reductions in the KRAS transcript levels in the tested concentration range (0.1–10 μM), while a scrambled ASO did not result in apparent knockdown. The antisense activity was in the order: ASO2 > ASO1 > ASO3. The downregulation of KRAS mRNA by ASO1 and ASO3 is likely mediated by RNase H1, an endonuclease that degrades the RNA of an RNA/DNA heterocomplex.^47^ When RNase H1 was decreased by an siRNA for 3 days in NCI-H358 cells (Fig. 3B), subsequent treatment with Lipo-complexed PS ASO1 and PS ASO3 resulted in less effective KRAS mRNA downregulation (Fig. 3C and Fig. S3A, ESI†). However, treatment with Lipo-PS ASO2 in RNase H1-deficient or normal NCI-H358 cells showed comparable KRAS mRNA levels (Fig. S3B, ESI†), suggesting that PS ASO2 may not work *via* an RNase H1-dependent mechanism.

**Fig. 3:**
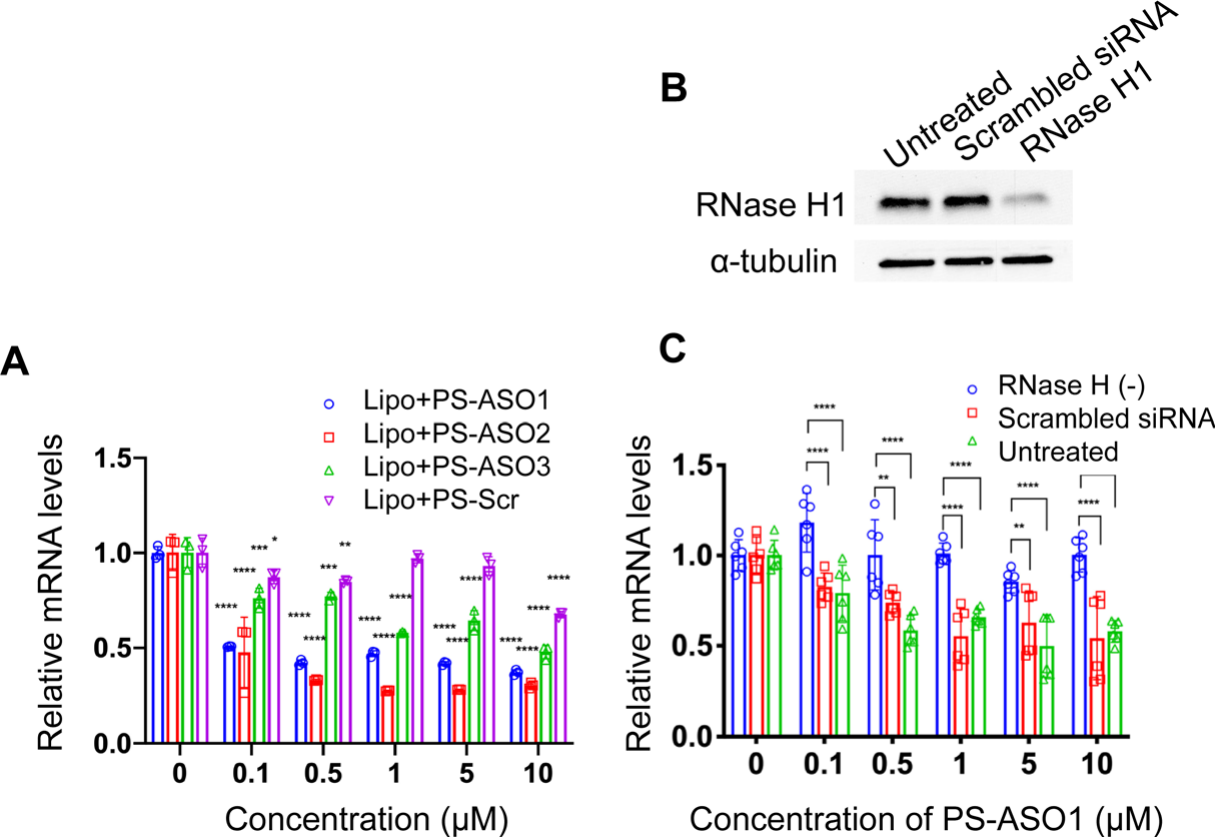
(A) Lipofectamine2k-assisted transfection of PS ASOs for 24 h causes significant sequence-dependent reduction in KRAS mRNA levels in NCI-H358 cells. (B) Western blot of NCI-H358 cell lysates after cells were treated with siRNA to knockdown RNase H1. (C) Reduction of RNase H1 eliminates the gene knockdown effect of PS ASO1, suggesting that free PS ASO1 depends on RNase H1 for target mRNA degradation. qPCR results are shown in mean ± standard deviation from at least three individual experiments. All results are normalized to β-actin mRNA. *P < 0.05, **P < 0.01, ***P < 0.001, and ****P < 0.0001 (one-way ANOVA, Tukey's test).

Next, pacDNA formulations of the ASOs 1–3 (named correspondingly pacDNA 1–3) were prepared and tested. We first screened the pacDNAs for inhibition of cellular growth (Fig. S4A, ESI†). Despite that ASO-2 was the most effective in lowering KRAS mRNA levels, PO pacDNA-2 did not effectively inhibit NCI-H358 cell proliferation. Western blotting indicates only ∼8% reduction in protein expression at 10 μM (Fig. S4B, ESI†). For MIA PaCa-2 cells, which harbors the KRAS G12C mutation, PO pacDNA-2 was also not as effective as the PO pacDNA-1 in inhibiting cell growth (Fig. S4C, ESI†), with KRAS reduced by 16% at 10 μM (Fig. S4D, ESI†). We therefore focused on ASO1 and ASO3 in subsequent mechanistic studies, which target the mutation site and the 3′-UTR, respectively.^48,49^ Next, NCI-H358 cells were treated with the PO pacDNA-1 and PO pacDNA-3 for 24 h and 48 h. No significant reduction of KRAS mRNA was detected at either time point by qRT-PCR (Fig. 4A). Expectedly, knocking down RNase H1 beforehand had no effect on KRAS mRNA levels in response to pacDNA treatment (Fig. 4B). Similarly, PS pacDNA-1 and PS pacDNA-3 did not reduce KRAS mRNA levels (Fig. S5A, ESI†) but showed significant inhibition on cell proliferation (Fig. S5B, ESI†). These results suggest that the pacDNA does not significantly affect the mRNA level of the target gene, despite the ASO being RNase H-acceptable. However, KRAS protein levels were notably reduced in a dose-dependent manner when cells were treated with both PS and PO forms of pacDNAs for 72 h (Fig. 5), while scrambled pacDNA controls (pacDNA-scr in both PO and PS forms) did not lead to KRAS downregulation. Knockdown efficiency was between 17% and 40% at 1 μM ASO concentration as determined by densitometry analysis of western blots. Interestingly, although Lipo-PS ASO1 was able to significantly reduce KRAS mRNA transcript levels, its ability to affect KRAS protein levels is comparable to PS pacDNA-1. These results suggest that pacDNA in general inhibits protein expression *via* the steric block mechanism, which may occur at multiple points during the translation process including disruption of the regulatory functions of 3′ and 5′ UTR, prevention of ribosome assembly, or steric blockade of the start codon/coding sequence. The observation that pacDNAs with PO or PS ASOs do not exhibit RNase H-dependent mRNA cleavage may be attributed to the bottlebrush component, which blocks RNase H from accessing the mRNA/ASO heteroduplex.

**Fig. 4:**
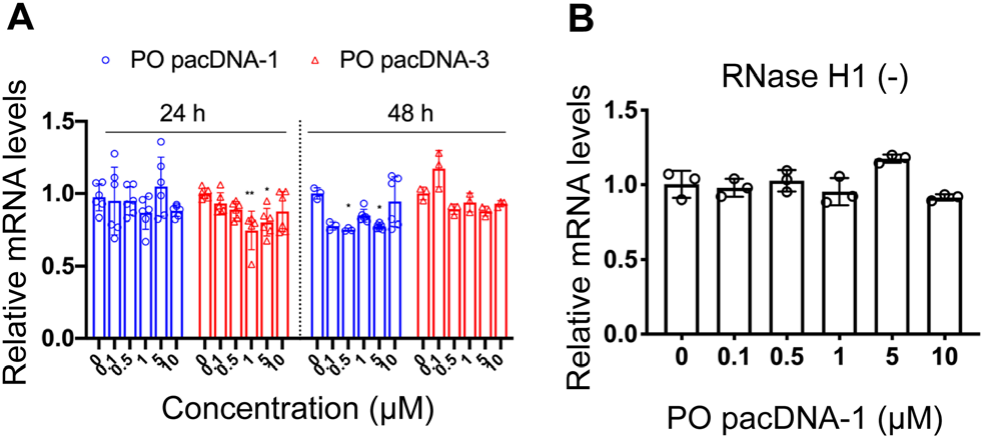
(A) Relative KRAS mRNA levels upon treatment with pacDNA containing different ASOs for 24 h or 48 h, showing no significant down-regulation. (B) Pre-knockdown of RNase H1 has no effect on KRAS mRNA levels following incubation with PO pacDNA-1 for 24 h. The qRT-PCR results are shown in mean ± standard deviation from at least three individual experiments. All results are normalized to β-actin mRNA. *P < 0.05, **P < 0.01 (one-way ANOVA, Tukey's test).

**Fig. 5:**
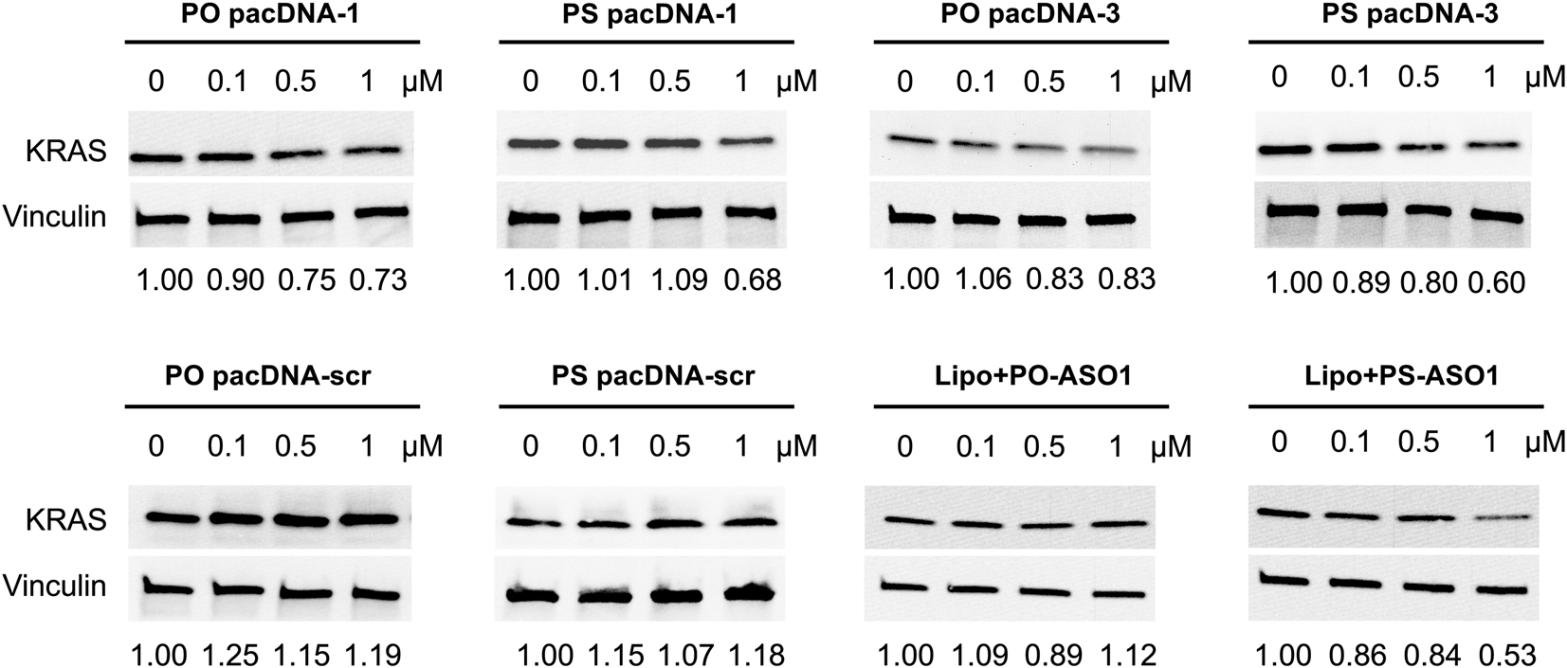
Western blot of NCI-H358 cell lysates after cells were incubated with pacDNA samples and controls at a concentration range of 0–1.0 μM for 72 h. The relative KRAS protein expression levels are shown below the blot images after normalization to vinculin.

One implication of this mechanism is that the gene knockdown for pacDNA becomes independent of ASO chemistry. To test this hypothesis, we furnished the pacDNA with a fully 2′-*O*-methyl-modified ASO1 (OMe pacDNA-1). This modification is known to be less toxic than PS and has higher affinity for their target sequence but is more RNA-like and cannot induce RNase H-dependent degradation. Indeed, Lipo-assisted transfection using PO OMe ASO1 did not reduce the KRAS mRNA levels, neither did OMe pacDNA-1 (Fig. 6A and B). With western blotting, however, significant reduction (41%) of KRAS protein expression was observed in cells treated with OMe pacDNA-1 at 1 μM ASO for 72 h (Fig. 6C). The reduction in KRAS protein levels is associated with retarded cell proliferation (Fig. 6D). Similarly, pacDNA made with the locked nucleic acid (LNA) form of ASO1 (LNA pacDNA-1) did not reduce the KRAS mRNA levels (Fig. S6A, ESI†), but reduced the KRAS protein expression by 33% at 1 μM ASO (Fig. S6B, ESI†) and inhibited cell proliferation to a greater extent than PO pacASO-1 (Fig. S6C, ESI†).

**Fig. 6:**
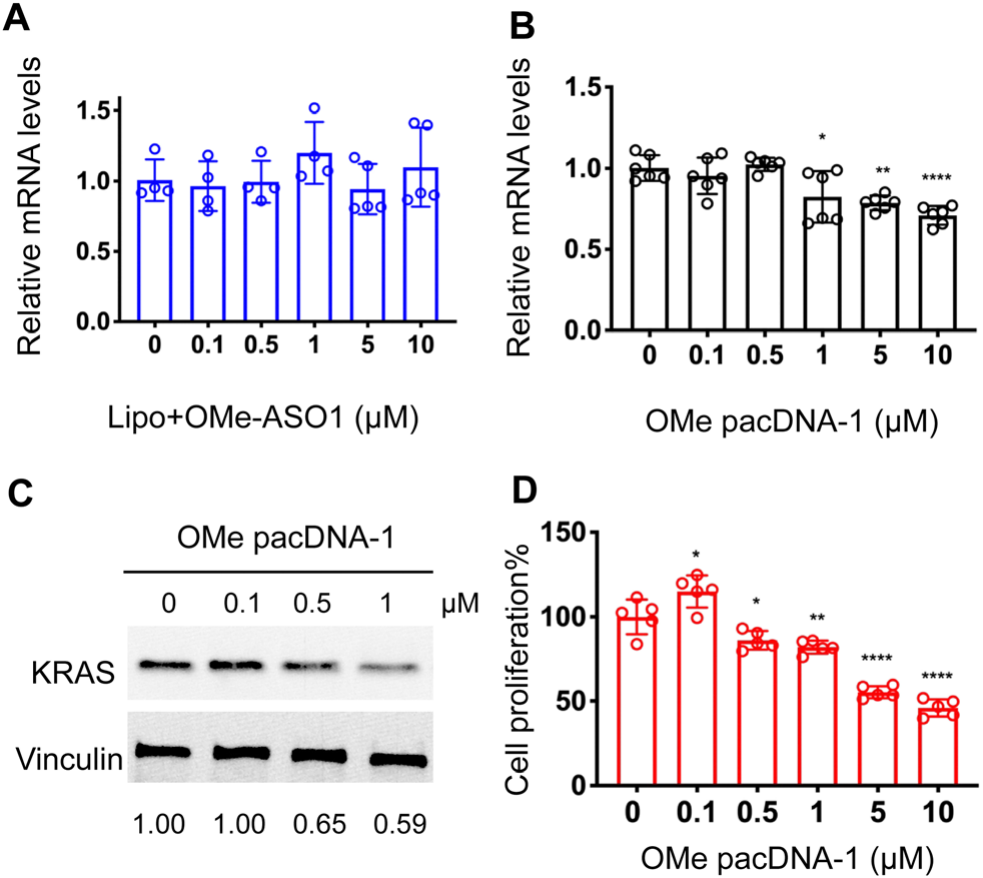
(A and B) Both Lipofectamine2k-assisted transfection of OMe ASO1 and unformulated OMe pacDNA-1 do not reduce KRAS mRNA levels. The qRT-PCR results are shown in mean ± standard deviation from at least three individual experiments. All results are normalized to β-actin mRNA. *P < 0.05, **P < 0.01, ****P < 0.001 (one-way ANOVA, Tukey's test). (C) Western blot analysis of NCI-H358 cells after incubation with OMe pacDNA-1 at concentrations 0–1 μM for 72 h. The expression levels of KRAS normalized to vinculin are shown below the blot images. (D) Viability of NCI-H358 cells treated with OMe pacDNA-1 for 96 h as determined by an MTT assay. Error bars denote the standard deviation of five individual experiments.

## Conclusions

In summary, we demonstrate that the pacDNA construct enters NCI-H358 cells predominantly *via* SR-A-mediated endocytosis and macropinocytosis. After endolysosomal trafficking, it is speculated that a fraction of the pacDNA has gained access to the cytosol, where it causes translational arrest of the target mRNA by steric blocking. In NCI-H358 cells, the activity of the pacDNA is not dependent on the ASO chemistry; RNase H-inactive ASO modifications can still cause target gene downregulation as long as they retain target binding affinity. We anticipate the fundamental understanding of how the pacDNA functions *in vitro* will provide the foundation for more in-depth mechanistic explorations and disease-specific structural optimizations of the pacDNA.

## Author contributions

K. Z. and L. Z. devised the experiments and wrote the manuscript. L. Z. and Y. W. conducted the synthesis of materials, purification, and material/biological characterizations. All other authors contributed to material synthesis, purification, molecular dynamics simulation, and/or discussion of the results. All authors edited the manuscript.

## Conflicts of interest

K. Z. has financial interest in PACDNA LLC.

## Supplementary Material

CB-004-D2CB00149G-s001
