# Supplementary material for: A mechanistic study on the cellular uptake, intracellular trafficking, and antisense gene regulation of bottlebrush polymer-conjugated oligonucleotides
Source: RSC Chem Biol. 2022 Nov 8;4(2):138–45. doi: 10.1039/d2cb00149g (PMC9906284; doi:10.1039/d2cb00149g)
Supplement: CB-004-D2CB00149G-s001 [file CB-004-D2CB00149G-s001.pdf]

Electronic Supplementary Material (ESI) for RSC Chemical Biology.
This journal is © The Royal Society of Chemistry 2022

Supplementary Information

A Mechanistic Study on the Cellular Uptake, Intracellular Trafficking, and Antisense Gene Regulation of Bottlebrush Polymer-Conjugated Oligonucleotides

Lei Zhang[a,b,†], Yuyan Wang[b,†], Peiru Chen[b], Dali Wang[b], Tingyu Sun[b], Zheyu Zhang[b], Ruimeng Wang[b], Xi Kang[b], Yang Fang[b], Hao Lu[b], Jiansong Cai[b], Mengqi Ren[b], Sijia S. Dong[b],*, and Ke Zhang[c],*

[a]Chemicobiology and Functional Materials Institute, School of Chemistry and Chemical Engineering, Nanjing University of Science and Technology, Nanjing 210094, P.R. China
[b]Department of Chemistry and Chemical Biology, Northeastern University, Boston, Massachusetts 20115, United States
[c]Departments of Chemistry and Chemical Biology, Chemical Engineering, and Bioengineering, Northeastern University, Boston, Massachusetts 20115, United States
[†]These authors contributed equally.

Materials. ω-Amine PEG methyl ether (Mn=10 kDa, PDI=1.05) was purchased from JenKem Technology, USA. Dibenzocyclooctyne-N-hydroxysuccinimidyl ester (DBCO-NHS) was purchased from Sigma-Aldrich Co., USA. Phosphoramidites and supplies for DNA synthesis were obtained from Glen Research Co., USA. NCI-H358 human non-small cell lung cancer cell line was purchased from American Type Culture Collection (Rockville, MD, USA). All other materials were obtained from Fisher Scientific Inc., USA, or VWR International LLC., USA, and used as received unless otherwise indicated.

Instrumentation. *N*,*N*-dimethylformamide (DMF) gel permeation chromatography (GPC) was performed on a TOSOH EcoSEC HLC-8320 GPC system (Tokyo, Japan) equipped with a TSKGel GMHHR-H, 7.8×300 mm column and RI/UV-Vis detectors. HPLC-grade DMF with 0.05 M LiBr was used as the mobile phase, and samples were run at a flow rate of 0.4 mL/min. GPC calibration was based on polystyrene standards (706 kDa, 96.4 kDa, 5970 Da, 500 Da). Aqueous GPC measurements were carried out on a Waters Breeze 2 GPC system equipped with an Ultrahydrogel™ 1000, 7.8×300 mm column and three Ultrahydrogel™ 250, 7.8×300 mm columns and a 2998 PDA detector for the separation of as-synthesized polymers from monomers/oligonucleotides. Sodium nitrate solution (0.1 M) was used as the eluent running at a flow rate of 0.8 mL/min. To purify the oligonucleotides, reversed-phase HPLC was performed on a Waters (Waters Co., MA, USA) Breeze 2 HPLC system coupled to a Symmetry® C18 3.5 μm, 4.6×75 mm reversed-phase column and a 2998 PDA detector, using TEAA buffer (0.1 M) and HPLC-grade acetonitrile as mobile phases. MALDI-TOF MS measurements were performed on a Bruker Microflex LT mass spectrometer (Bruker Daltonics Inc., MA, USA).

Oligonucleotide synthesis. Oligonucleotides including modifications were synthesized on a Model 391 DNA synthesizer (Applied Biosystems, Inc., Foster City, CA) using standard solid-phase phosphoramidite methodology. DNA were cleaved from the CPG support using aqueous ammonium hydroxide (30% NH_3 basis) at room temperature for 18 h. OMe-modified strands were synthesized on Universal Support III PS CPG, cleaved by treating with 2 M ammonia in methanol at room temperature for 60 minutes, and deprotected using aqueous ammonium hydroxide (30% NH_3 basis) at room temperature for 18 h. All strands were purified by reversed-phase HPLC. The successful synthesis of all sequences was confirmed by MALDI-TOF MS.

Synthesis of bottlebrush polymer. The diblock bottlebrush polymer (**3**, Scheme S1) was synthesized via ring-opening polymerization of norbornenyl bromide (**1**) and norbornenyl PEG (**2**), following by azide substitution. The synthesis of norbornenyl bromide, norbornenyl PEG, and modified 2nd generation Grubbs' catalyst has been described in our previous publications.[1-2] Typically, a solution of norbornenyl bromide (**1**, 5 equiv.) in deoxygenated dichloromethane was added into a Schlenk flask under N_2. The solution was cooled to -20 °C in an ice-salt bath, to which modified Grubbs' catalyst (1 equiv.) in deoxygenated dichloromethane was added. The reaction mixture was stirred vigorously for 30 min until thin-layer chromatography (TLC) confirmed the complete consumption of the monomer. Then, a solution of **2** (30 equiv.) in deoxygenated dichloromethane was added to the reaction. The reaction mixture was further stirred for 6 h, before addition of several drops of ethyl vinyl ether (EVE) to remove the chain-end catalyst. Note that all solution was added by gastight syringe to reduce the amount of oxygen introduced to the reaction. The mixture was stirred overnight, and the product was precipitated into cold diethyl ether 3× and dried under vacuum. The as-synthesized polymer was treated with an excess of sodium azide in DMF overnight at room temperature to give the azide-functionalized bottlebrush polymer, **3**. The product was dialyzed against Nanopure™ water for 24 h, lyophilized, re-dissolved in Nanopure™ water, and injected into an aqueous GPC for collection of the fractions containing the bottlebrush polymer. The final polymer was desalted using a NAP-10 column (G.E. Healthcare, IL, USA). DMF-GPC analysis determines the bottlebrush polymer with Mn = 290 kDa, Mw = 390 kDa, PDI = 1.34.

Synthesis of pacDNA. In a typical procedure, azide-functionalized bottlebrush polymer **3** (50 nmol) was dissolved in 100 μL Nanopure™ water, to which DBCO-modified DNA (or chemically modified forms) was added (2.2 equiv. to N_3, 50 μL aqueous solution). The reaction mixtures were shaken gently for 24 h at 50 °C on an Eppendorf Thermomixer. Thereafter, aqueous GPC was used to isolate the conjugation product from unreacted DNA. The conjugates were desalted using a NAP-10 column and lyophilized to yield a white powder (or red powders for Cy3-labeled pacDNA).

Synthesis of dual-labeled pacDNA. To simultaneously label the bottlebrush polymer and DNA strands for *in vitro* fluorescence tracking, the azide-functionalized polymer was first labelled with Cy5 before coupling to Cy3-labeled DNA. Specifically, polymer **3** (50 nmol) and alkyne-modified Cy5 (150 nmol, 150 μL of 1 mM stock solution in DMSO) were mixed in Nanopure™ water (2 mL), followed by the addition of the catalyst system ($CuSO_4 \cdot 5H_2O$, 40

nmol; tris(3-hydroxypropyltriazolylmethyl)amine known as THPTA, 50 nmol; sodium ascorbate, 250 nmol). After 12 hours of stirring, the reaction mixture was dialyzed against Nanopure™ water to remove small molecular residuals and lyophilized. The Cy5-labeled bottlebrush polymer was then coupled to Cy3-conjugated DNA under the same conditions as described above, purified by aqueous GPC, desalted, and lyophilized to yield a purple powder.

Cell culture. NCI-H358 and MIA PaCa-2 cells were cultured in RPMI-1640 medium supplied with 10% fetal bovine serum (FBS, Gibco, USA), 100 units/mL of penicillin and 100 μg/mL of streptomycin (Gibco, USA) at 37 °C in a humidified atmosphere containing 5% CO_2.

Cellular uptake. Cellular uptake kinetics was evaluated using flow cytometry. Cells were seeded in 24-well plates at 3.0×10^5 cells per well in 1 mL complete RPMI-1640 medium and cultured for 24 h at 37 °C with 5% CO_2. pacDNA-3-Cy3 (2 μM equiv. of DNA) was dissolved in RPMI culture medium containing 10% FBS, and cells were further incubated at 37 °C for 10 min through 48 h. In a parallel study, cells were incubated with pacDNA in RPMI culture medium without FBS. At predetermined time points, cells were washed with PBS before being treated with trypsin-EDTA (Gibco, USA). Thereafter, 2 mL of PBS was added to each culture well, and the solutions were centrifugated for 5 min (1000 rpm). Cells were then resuspended in 0.5 mL of PBS for flow cytometry analysis on an Attune NxT flow cytometer (Thermo Fisher Scientific, USA). The fluorescence signals were collected using 455 nm as the excitation laser, with the BL2 channel (574/26 nm) as the emission filter. For each measurement, 3.0×10^4 gated events were collected. The experiments were carried out in triplicates.

For confocal microscopy, cells were seeded on a glass slide of 12 mm in diameter (Marienfeld Superior) in a 24-well plate (Fisher Scientific, USA) at 3×10^5 cells per well and cultured for 24 h. The dual-labeled Cy5-pacDNA-3-Cy3 mixed with 0.5 mL full medium (2 μM equiv. of DNA) was added, and the cells were further incubated for 4 h or 24 h. Cells were then washed with PBS, fixed with 4% paraformaldehyde (PFA), and counter-stained with Hoechst 33342 for 10 min. Cells were washed with PBS 3×, mounted to the glass slides with Fluoromount-G™ mounting medium (Thermo Fisher Scientific, USA), and dried overnight. The samples were then imaged on an LSM-700 confocal laser scanning microscope (Carl Zeiss Ltd., Cambridge, UK). Images were taken at excitation wavelengths of 350 nm (Hoechst 33342), 532 nm (Alexa Fluor 488), and 640 nm (Alexa Fluor 488). To quantify the colocalization of the two fluorescence signals of pacDNA, Image J (Fiji contributors) with a Coloc2 plugin was

used to calculate the Manders colocalization coefficient according to the protocols provided by the software authors, where G represents green pixel signals (pseudocolor for Cy3) and R represents red pixel signals (Cy5). The threshold value was kept identical for all images analysed and the M1 coefficient was used for comparisons.

$$M1 = \frac{\sum_i G_i, colocal}{\sum_i G_i} \quad M2 = \frac{\sum_i R_i, colocal}{\sum_i R_i}$$

Pharmacological inhibition. Cells were seeded in 24-well plates at 3×10^5 cells per well overnight before being pre-treated with 300 µL of RPMI-1640 medium that contains different concentrations of chemical blockers for 1 h (Table S2). These blockers include fucoidan (50 µg/mL, Sigma Aldrich Fine Chemicals Biosciences), filipin III (2.5 µg/mL, Cayman Chemical), chlorpromazine (5 µg/mL, Tokyo Chemical Industry), amiloride (500 µg/mL, Cayman Chemical), dynasore (120 µM, MilliporeSigma™), and methyl-β-cyclodextrin (M-β-CD) (10 mM, Sigma-Aldrich). After removing the inhibitor-containing medium, 0.5 mL of serum-containing medium or sole RMPI-1640 medium that contains the same inhibitor at the original concentration and 2 µM of pacDNA-3-Cy3 were subsequently added to the cells. After 4 h of incubation, the medium was removed, and the cells were rinsed with PBS and trypsinized. Cells were then resuspended in 0.5 mL of PBS for flow cytometry analysis on an Attune NxT flow cytometer (Thermo Fisher Scientific, USA). The Cy3 fluorescence signals were collected using blue laser (455 nm) as the excitation light with BL2 channel (574/26 nm) as the emission filter. For each measurement, 3.0×10^4 gated events were collected. The experiments were carried out in triplicates.

Immunofluorescence staining of cells. Cells were seeded on a glass slide of 12 mm in diameter (Marienfeld Superior) in a 24-well plate (Fisher Scientific, USA) at 3×10^5 cells per well overnight before being incubated with 0.5 mL FBS-containing medium containing 2 µM of pacDNA-3-Cy3 for 1 h through 32 h. In a parallel study, 2 µM of pacDNA-3-Cy3 in RPMI-1640 medium without FBS was used. After treatment, cells were washed, fixed with 4% paraformaldehyde (PFA), and permeated with 1% Triton X-100 (Sigma-Aldrich) for 10 min. Cells were blocked with 1% BSA in PBS for 1 h at room temperature, before being stained with primary antibodies (in PBS with 1% BSA) overnight at 4 °C. The primary antibodies include those antibodies against EEA1 (Invitrogen, Cat. No. MA5-14794) (1:500), Rab9

(Invitrogen, Cat. No. MA3-067) (1:500), and Lamp1 (Abcam, Cat. No. ab24170) (1:1000). After rinsing with 0.05% Tween-20 in PBS, cells were stained with Alexa Fluor 488-conjugated goat anti-rabbit IgG (H+L) secondary antibody (Invitrogen, Cat. No. A11008) (for EEA1 and Lamp1), or Alexa Fluor 488-conjugated goat anti-mouse IgG secondary antibody (Invitrogen, Cat. No. A11029) at 1 μg/mL (1% BSA in PBS) for 1 h at room temperature. Cells were counter-stained with Hoechst 33342 for 10 min, washed with PBS, mounted to the glass slides with Fluoromount-G™ mounting medium (Thermo Fisher Scientific, USA), and dried overnight. The samples were then imaged on an LSM-700 confocal laser scanning microscope (Carl Zeiss Ltd., Cambridge, UK). Images were taken at excitation wavelengths of 350 nm (Hoechst 33342) and 488 nm (Alexa Fluor 488). To quantify the colocalization between fluorescence signals of pacDNA and cellular compartments, Image J (Fiji contributors) with a Coloc2 plugin was used to calculate the Manders colocalization coefficient according to the protocols provided by the authors, where R represents red pixel signals (Cy3) and G represents green pixel signals (Alexa Fluor 488). Manders' tM2 (above autothreshold of red channel) coefficient was use to indicate the degree of co-localization.

MTT assay. The cell proliferation inhibition effect of pacDNA was evaluated with the MTT assay against NCI-H358 cells. Briefly, NCI-H358 cells were seeded into 96-well plates at 2.5×10^4 cells per well in 200 μL full medium and cultured for 24 h. The cells were then treated with pacDNA at varying concentrations of DNA (0.1 through 10 μM). Cells treated with vehicle (PBS) were used as a negative control. After 96 h of incubation, 20 μL of 5 mg/mL MTT stock solution in PBS was added to each well. The cells were incubated for another 4 h, and the medium containing unreacted MTT was removed carefully. The resulting blue formazan crystals were dissolved in 200 μL DMSO per well, and the absorbances (490 nm) were measured on a BioTek® SynergyTM Neo2 Multi-Mode microplate reader (BioTek Inc., VT, USA).

Quantification of mRNA levels by qRT-PCR. NCI-H358 cells were plated at a density of 3.0×10^5 cells per well in 24-well plates and cultured overnight. Cells were incubated with pacDNA (0.1 through 10 μM), or Lipofectamine2k-complexed DNA (0.1 through 10 μM) in full medium for specified durations of time. Lipofectamine2k was first mixed with PS ASOs in serum-free medium for 15 min before addition to full medium at a concentration of 1 μg/mL. A two-step qRT-PCR method was used to quantify KRAS mRNA levels. After incubation, the total RNA was extracted using the Trizol reagent (Invitrogen, Thermo Fisher Scientific)

following manufacture-suggested protocols. The RNA concentration was determined using a NanoDrop 2000 spectrophotometer (Thermo Scientific). Total RNA (300 ng) was reverse-transcribed to cDNA using the SuperRT cDNA Synthesis kit (CWBIO) at 42 °C for 30 min and 85 °C for 5 min. The cDNA was amplified with SsoAdvanced Universal SYBR Green Super Mix on a Bio-Rad CFX96 Touch System. The results were normalized to β-actin expression. The primer sequences used were: KRAS forward-GAC ACA AAA CAG GCT CAG GAC TT, reverse-TCT TGT CTT TGC TGA TGT TTC AAT AA; β-actin forward- CGG ACT ATG ACT TAG TTG CGT TAC A, reverse-GCC ATG CCA ATC TCA TCT TGT. A second primer set was used to verify the result by normalization to GAPDH expression: KRAS forward-GCC TGC TGA AAA TGA CTG AAT ATA, reverse-TTA GCT GTA TCG TCA AGG CAC TC, GAPDH forward-AAT CCC ATC ACC ATC TTC CA, reverse-TGG ACT CCA CGA CGT ACT CA. All qRT-PCR experiments were performed at least in triplicates and the results were averaged. Fold difference in relative changes in gene expression is according to the $2^{-\Delta\Delta Ct}$ formula as described by Livak et al.[3]

Western blotting. Cells were seeded at a density of 2.0×10^5 cells in a 24-well plate overnight, and incubated with pacDNA or Lipofectamine2k-complexed DNA in serum-containing medium for 72 h. Thereafter, whole-cell lysates were collected in 100 μL of RIPA lysis and extraction buffer (Thermo Scientific, USA) containing Halt™ protease and phosphatase inhibitor cocktail (Thermo Scientific, USA) following manufacturer's protocols. Protein content in the extracts was quantified using a bicinchoninic acid protein assay kit (Thermo Fisher Scientific, MA, USA). Equal amounts of proteins (20 μg per lane) were separated on 4-20% gradient SDS–polyacrylamide gel electrophoresis and electro-transferred to a nitrocellulose membrane. The membranes were then blocked with 3% bovine serum albumin in tris-buffered saline supplemented with 0.05% Tween 20 (TBST) and further incubated with primary antibodies at 4°C overnight, including KRAS antibody (Cat. No. NBP2-45536, Novus Biologicals, CO, USA) (1:1000 dilution), phospho-p44/42 MAPK (Erk1/2) (Thr202/Tyr204) antibody (Cell Signalling Technology, MA, USA) (1:1000 dilution), and vinculin monoclonal antibody, clone hVIN-1 (Cat. No. V9131, Sigma Aldrich, USA) (1:200 dilution). After washing with TBST 3×, membranes were further incubated with anti-mouse IgG, HRP-linked secondary antibody (Cell Signalling Technology, MA, USA) (1:2000 dilution). Protein bands were visualized by chemiluminescence using the ECL Western Blotting Substrate (Thermo Fisher Scientific, MA, USA).

Gene regulation in cells with knocked-down RNase H1. NCI-H358 cells were seeded in 24-well plates for 12 h before transfection. The transfection medium was formulated in serum-free medium containing 5 µg/mL of Lipofectamine2k (Invitrogen) and either 200 nM of mouse small-interfering RNA (siRNA) that specifically targets the human RNaseH1 (SMARTpool, Dharmacon) or 200 nM of nontargeting control siRNA (siControl) (Dharmacon). After 4 h of transfection, the transfection medium was switched to full medium for 20 h. The cells were then cultured in RPMI-1640 medium supplemented with 0.5% FBS and 1% penicillin-streptomycin for another 48 h. Thereafter, cells were incubated with full medium and pacDNA or Lipofectamine2k-complexed PS ASOs for 6 h. Cells were harvested and digested for western blotting as indicated above. The primary antibody used was first incubated with RNase H1 polycolonal antibody (Cat. No.156061AP, Proteintech Group Inc.) (1:1000 dilution), and visualized by incubating with anti-rabbit IgG, HRP-linked secondary antibody (Cell Signaling Technology, MA, USA) (1:2000 dilution).

Coarse-grained molecular dynamics simulation. An all-atom structure of pacDNA was mapped to coarse-grained (CG) beads according to functional groups that best match the bead types in the MARTINI force field[4] (2-5 atoms per bead). The CG parameters for the polymer backbone and linkers were extracted from a molecular dynamics (MD) trajectory of an atomistic simulation of a three-repeating unit model molecule based on the OPLS-AA force field.[5] The coarse-grained structure was solvated in a CG water box. Sodium ions were added to ensure the system is neutral in charge. The solvated system underwent energy minimization, followed by 50 ns of equilibration and 10 ns of production MD simulation (step size: 4 fs; NPT ensemble) using GROMACS 2021.3[6] with the velocity rescale thermostat[7] and the Parrinello-Rahman barostat[8] under 300 K and 1 bar.

Scheme S1. Chemical synthesis of pacDNA.

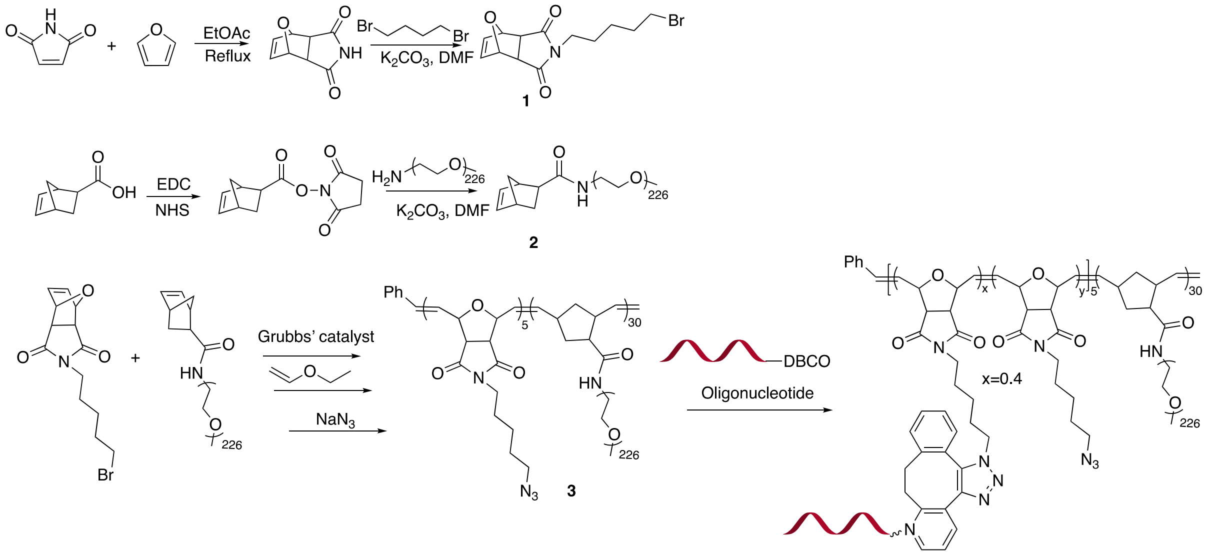

Scheme S2. Structure of dual-labeled pacDNA.

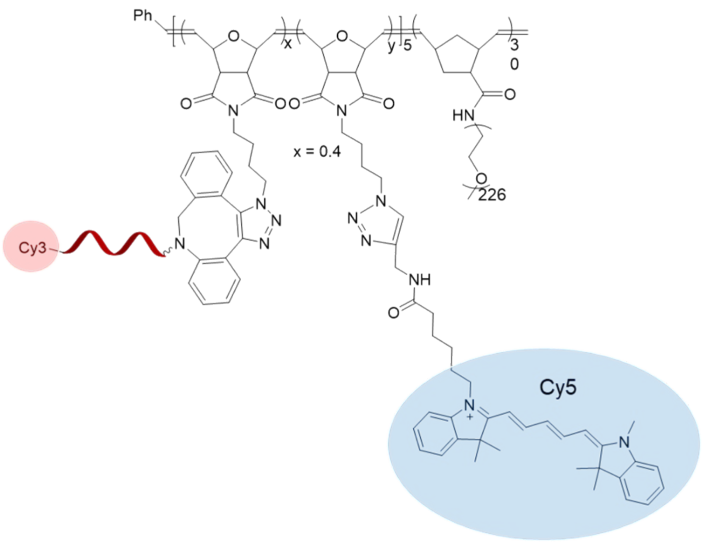

Table S1. Nomenclature of samples, ASO sequence, and assay performed.

Sample ID	Assay	ASO sequence
**pacDNA-3-Cy3**	Cell uptake mechanism (flow cytometry); Intracellular trafficking (confocal microscopy)	5’-DBCO-GCT ATT AGG AGT CTT T-Cy3-3’ (**Cy3 ASO3**)
**Cy5-pacDNA-3-Cy3** (Cy5 labeling of polymer)	Intracellular stability (confocal microscopy)	5’-DBCO-GCT ATT AGG AGT CTT T-Cy3-3’ (**Cy3 ASO3**)
**Lipo+PS-1** (Lipofectamine2k-ASO complex)	mRNA expression (qRT-PCR)	5’-cta cgc cac aag ctc ca-3’ (**PS ASO1**)
**Lipo+PS-2** (Lipofectamine2k-ASO complex)		5’-gca ctg tac tcc tct t-3’ (**PS ASO2**)
**Lipo+PS-3** (Lipofectamine2k-ASO complex)		5’-gct att agg agt ctt t-3’ (**PS ASO3**)
**Lipo+PS-Scr** (Lipofectamine2k-ASO complex)		5’-gcc atc gca cca act ac-3’ (**PS-Scr**)
**PO pacDNA-1**	mRNA expression (qRT-PCR) (**PO pacDNA-1**); Cell proliferation (MTT assay); KRAS protein expression (Western blotting)	5’-DBCO-CTA CGC CAC AAG CTC CA-3’ (**PO ASO1**)
**PO pacDNA-2**		5’-DBCO-GCA CTG TAC TCC TCT T-3’ (**PO ASO2**)
**PO pacDNA-3**		5’-DBCO-GCT ATT AGG AGT CTT T-3’ (**PO ASO3**)
**PS pacDNA-1**	mRNA expression (qRT-PCR); Cell proliferation (MTT assay); KRAS protein expression (Western blotting)	5’-DBCO-cta cgc cac aag ctc ca-3’ (**PS ASO1**)
**PS pacDNA-3**		5’-DBCO- gct att agg agt ctt t-3’ (**PS ASO3**)
**OMe pacDNA-1**	mRNA expression (qRT-PCR); KRAS protein expression (Western blotting); Cell proliferation (MTT assay)	5’-DBCO-CTA CGC CAC AAG CTC CA-3’ (**OMe ASO1**)
**LNA pacDNA-1**		5’-DBCO-*CTA CGC CAC AAG CTC CA*-3’ (**LNA ASO1**)
**Lipo+PO OMe ASO1** (Lipofectamine2k-ASO complex)	mRNA expression (qRT-PCR)	5’-DBCO-CTA CGC CAC AAG CTC CA-3’ (**PS OMe ASO1**)

Upper case: PO DNA. Lower case: PS modification. Underline: 2'-*O*-methyl (OMe) modification. Italic: locked nucleic acid (LNA) modification.

Table S2. List of pharmacological inhibitors of pathways of endocytosis and receptors.

Target	Agent/condition	Stock solution	Final concentration
**Clathrin**	Chlorpromazine	1 mg/mL (in water)	5 µg/mL
**Energy-dependent endocytosis**	4 ^{o}C	-	-
**Lipid raft/caveolae**	Filipin III	2 mg/mL (in 100% DMSO), then dilute 1:7 in PBS	2.5 µg/mL
**SR-A receptor**	Fucoidan	10 mg/mL (in water)	100 µg/mL
**Macropinocytosis**	Amiloride	250 mg/mL (in 100% DMSO), then dilute 1:9 in water	250 µg/mL
**Dynamin**	Dynasore	80 mM (in 100% DMSO)	80 µM
**Lipid raft/caveolae**	M-β-CD	250 mg/mL (in water)	12.5 mg/mL

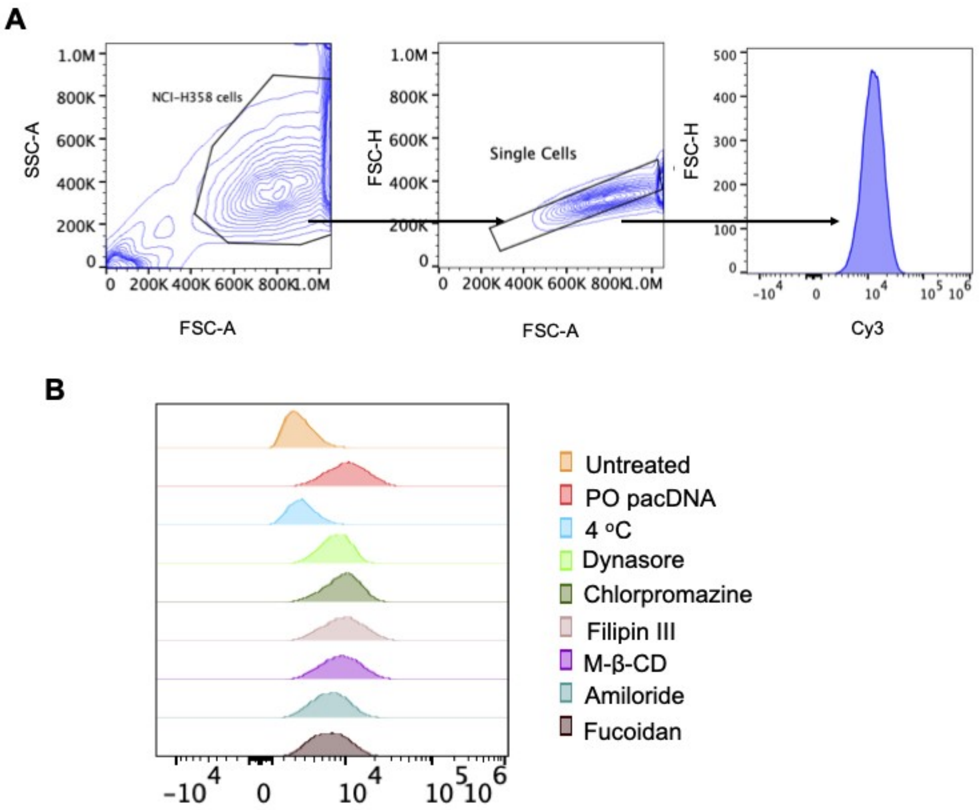

Figure S1. (A) Gating strategies for the uptake of Cy3-labeled PO pacDNA in NCI-H358 cells. (B) Overlay of the flow cytometry histograms showing the intensity of Cy3 after different treatments.

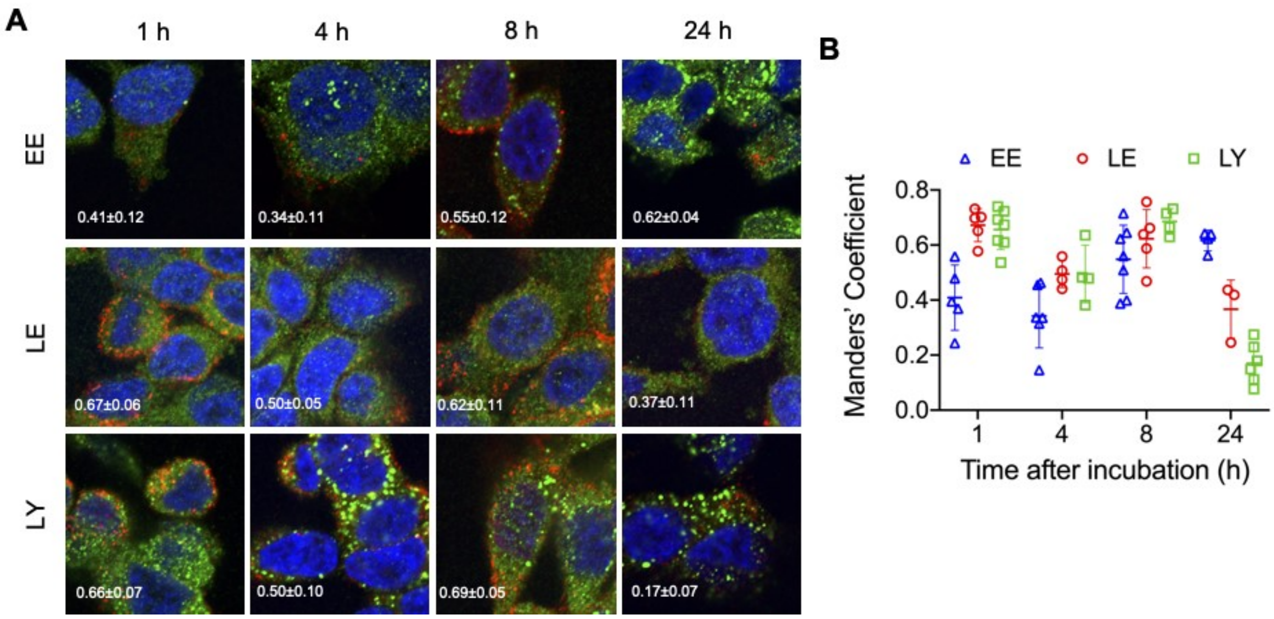

Figure S2. (A) Intracellular trafficking of pacDNA in NCI-H358 cells following different durations of incubation in serum-deprived medium. pacDNA: red; immunofluorescence staining of organelle markers: green. The markers include EEA1 (early endosome), Rab9 (late endosome), and LAMP1 (lysosome). Average Manders' colocalization coefficient with standard deviation (S.D.), shown in the bottom left of each image, of 0.5 or above indicates substantial colocalization. (B) Summary of the Manders' colocalization coefficient calculated from confocal images. In each treatment, over 20 cells in at least five images were analyzed, where each data point represents one image collected.

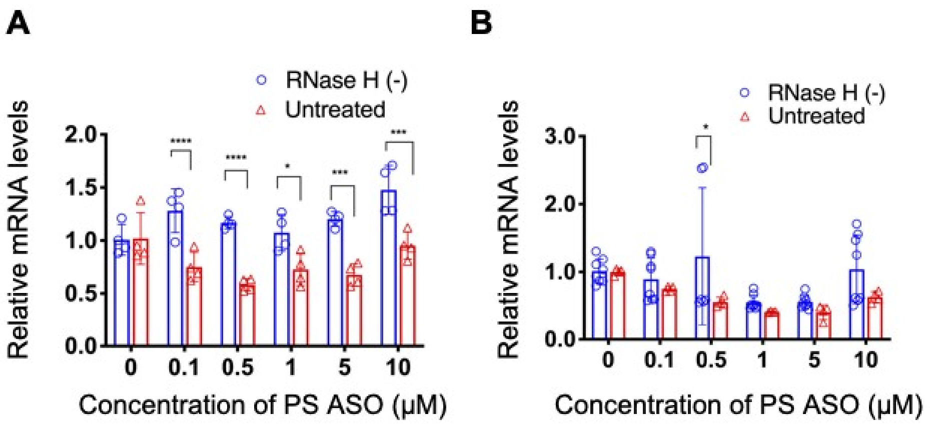

Figure S3. Correlation between the reduction in RNaseH1 expression and the KRAS knockdown in NCI-H358 cells. (A): Lipo-PS ASO3. (B): Lipo-PS ASO2. qPCR results are shown in mean ± standard deviation from at least three individual experiments. All results are normalized to β-actin mRNA. *P<0.05, ***P<0.001, and ****P<0.0001 (one-way ANOVA, Tukey's test).

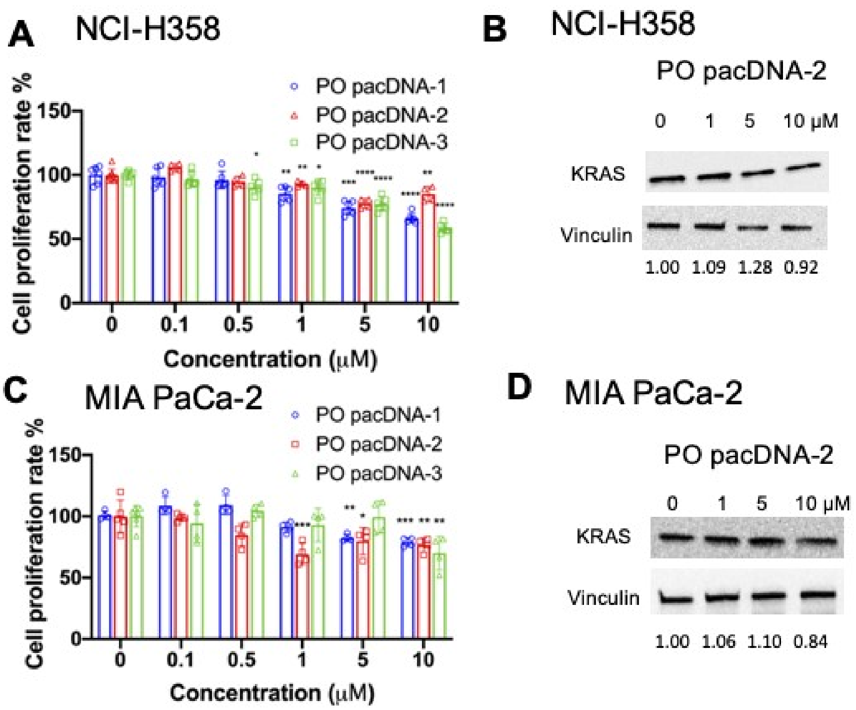

Figure S4. (A) Cell viability of NCI-H358 treated with pacDNA with different ASOs for 96 h as determined by an MTT assay. (B) Western blot of NCI-H358 cell lysates after cells were incubated with pacDNA samples and controls at a concentration range of 0-10 μM for 72 h. (C) Cell viability of MIA PaCa-2 cells treated with pacDNA with different ASOs for 96 h as determined by an MTT assay. (D) Western blot of MIA PaCa-2 cell lysates after cells were incubated with pacDNA samples and controls at a concentration range of 0-10 μM for 72 h. The relative KRAS protein expression levels are shown below the blot images after normalization to vinculin. In MTT assays, error bars denote the standard deviation resulting from four individual experiments.

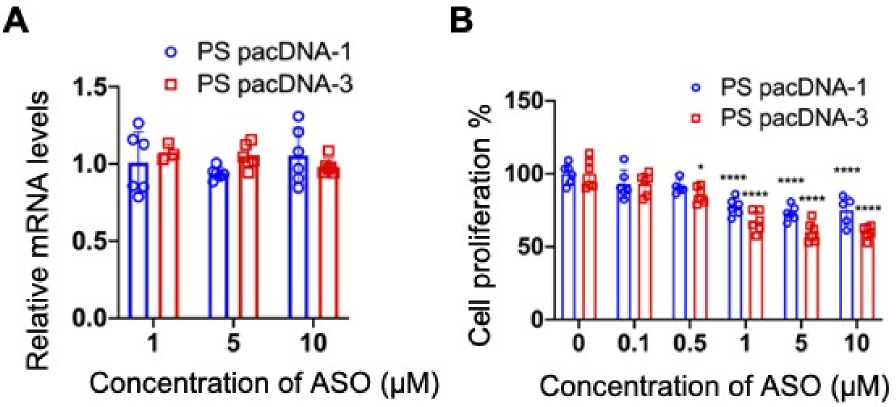

Figure S5. (A) Relative KRAS mRNA levels upon treatment with pacDNA containing ASO1 and ASO2 with full PS modifications for 24 h. qPCR results are shown in mean ± standard deviation from at least three individual experiments. All results are normalized to β-actin mRNA. (B) Cell viability of NCI-H358 treated with pacDNA containing ASO1 and ASO2 with full PS modifications for 96 h as determined by an MTT assay. *$*P<0.05$, and ****$P<0.0001$* (one-way ANOVA, Tukey's test).

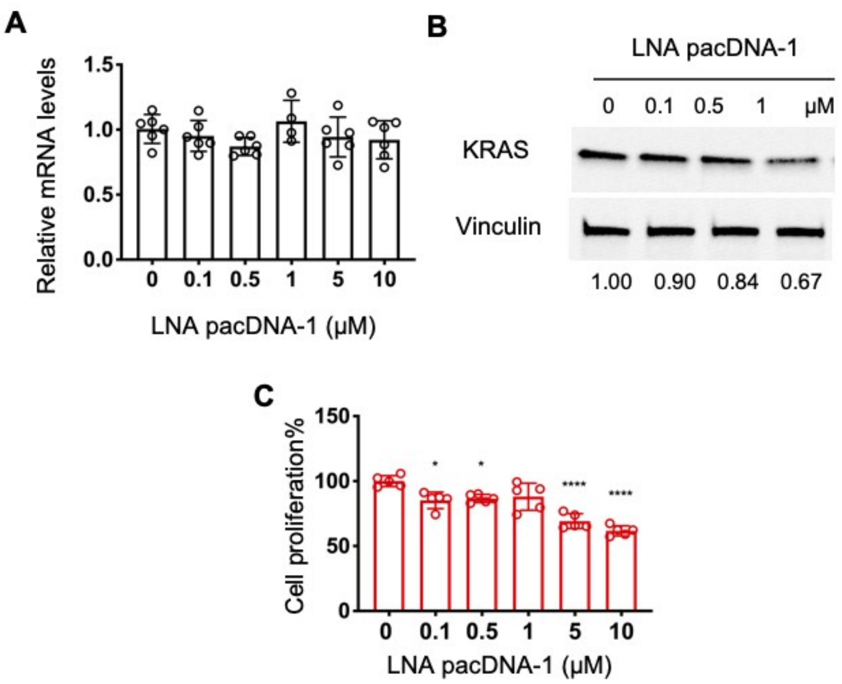

Figure S6. (A) LNA pacDNA-1 does not reduce KRAS mRNA levels. The qRT-PCR results are shown in mean ± standard deviation from at least three individual experiments. All results are normalized to β-actin mRNA. (B) Western blot analysis of NCI-H358 cells after incubation with LNA pacDNA-1 at concentrations 0-1 μM for 72 h. The expression levels of KRAS normalized to vinculin are shown below the blot images. (C) Viability of NCI-H358 cells treated with LNA pacDNA-1 for 96 h as determined by an MTT assay. Error bars denote the standard deviation of five individual experiments. **P*<0.05, and *****P*<0.001(one-way ANOVA, Tukey's test).

References

1. Lu, X.; Watts, E.; Jia, F.; Tan, X.; Zhang, K., Polycondensation of Polymer Brushes via DNA Hybridization. *J. Am. Chem. Soc.* **2014,** *136* (29), 10214-10217.
2. Wang, D.; Lin, J.; Jia, F.; Tan, X.; Wang, Y.; Sun, X.; Cao, X.; Che, F.; Lu, H.; Gao, X.; Shimkonis, J. C.; Nyoni, Z.; Lu, X.; Zhang, K., Bottlebrush-architectured poly(ethylene glycol) as an efficient vector for RNA interference in vivo. *Sci. Adv.* **2019,** *5* (2), eaav9322.
3. Livak, K. J.; Schmittgen, T. D., Analysis of Relative Gene Expression Data Using Real-Time Quantitative PCR and the 2−ΔΔCT Method. *Methods* **2001,** *25* (4), 402-408.
4. a) S. J. Marrink, H. J. Risselada, S. Yefimov, D. P. Tieleman, A. H. de Vries, *J. Phys. Chem. B* **2007**, *111*, 7812-7824; b) J. J. Uusitalo, H. I. Ingólfsson, P. Akhshi, D. P. Tieleman, S. J. Marrink, *J. Chem. Theory Comput.* **2015**, *11*, 3932-3945; c) F. Grunewald, G. Rossi, A. H. de Vries, S. J. Marrink, L. Monticelli, *J. Phys. Chem. B* **2018**, *122*, 7436-7449.
5. a) W. L. Jorgensen, D. S. Maxwell, J. Tirado-Rives, *J. Am. Chem. Soc.* **1996**, *118*, 11225-11236; b) L. S. Dodda, I. Cabeza de Vaca, J. Tirado-Rives, W. L. Jorgensen, *Nucleic Acids Res.* **2017**, *45*, W331-W336.
6. G. Bussi, D. Donadio, M. Parrinello, *J. Chem. Phys.* **2007**, *126*, 014101.
7. M. Parrinello, A. Rahman, *J. Appl. Phys.* **1981**, *52*, 7182-7190.
8. M. J. Abraham, T. Murtola, R. Schulz, S. Páll, J. C. Smith, B. Hess, E. Lindahl, *SoftwareX* **2015**, *1-2*, 19-25.
